# Willingness-to-pay to access Ingenol Mebutate Gel for Actinic Keratosis Treatment in the U.S. Setting

**DOI:** 10.36469/9879

**Published:** 2014-10-01

**Authors:** Michael Willis, Sandra Erntoft, Sofie Persson, Jenny M. Norlin, Ulf Persson

**Affiliations:** 1 The Swedish Institute for Health Economics (IHE), Lund, Sweden; 2 LEO Pharma A/S, Ballerup, Denmark; 3 The Swedish Institute for Health Economics (IHE), Lund, Sweden; Lund University, Department of Clinical Sciences, Health Economics Unit, Sweden; 4 The Swedish Institute for Health Economics (IHE), Lund, Sweden; LEO Pharma A/S, Ballerup, Denmark; 5 The Swedish Institute for Health Economics (IHE), Lund, Sweden; Lund University, Institute for Economic Research, School of Economics, Sweden

**Keywords:** willingness-to-pay, actinic keratosis, local skin reactions, patient preferences, contingent valuation, topical agents

## Abstract

**Background:** Currently available topical treatments for actinic keratosis (AK) adversely affect patients’ quality of life because of long treatment durations and long-lasting local skin reactions (LSRs), which may result in poor treatment adherence and patient outcomes. Ingenol mebutate gel, a recently introduced treatment for AK, is administered for 2 or 3 days, and LSR’s are predicable in onset and duration.

**Objectives:** The objective of the study was to estimate the value of ingenol mebutate gel’s shorter treatment duration and tolerability profile to potential patients, versus existing topical treatments (imiquimod 3.75%, imiquimod 5% and diclofenac 3%) in the United States.

**Methods:** The open-ended Contingent Valuation (CV) approach was used to estimate incremental willingness-to-pay (WTP) for ingenol mebutate gel rather than treatment with imiquimod 5%, imiquimod 3.75% and diclofenac 3%. Profiles for each therapy differed in regards to treatment duration, time-to-LSR resolution, and price. Subjects were asked to state their maximum out-of-pocket WTP to receive ingenol mebutate gel instead of each of the three alternatives.

**Results:** 103 subjects provided usable answers. Between 48% and 63% of subjects were willing to pay extra to gain access to treatment with the ingenol mebutate gel profile instead of the comparators, and the mean incremental WTP ranged from $475 to $518. Subjects with experience of topical treatment stated higher WTP for accessing ingenol mebutate gel. Subjects whose most bothersome AK area was the full scalp or forehead also claimed higher WTP for ingenol mebutate gel.

**Conclusions:** Patients diagnosed with AK indicated an unmet need for fast-acting topical treatment with shorter LSR resolution time.

## BACKGROUND

Actinic keratosis (AK), a precursor to non-melanoma skin cancer, is a skin condition consisting of thick, scaly, or crusty patches of lesions, often located in areas chronically exposed to the sun (such as the face, scalp, and hands) of fair-skinned, middle-aged and older individuals.[1] Studies suggest that 60-65% of squamous cell carcinoma (SCC) begin as AK[2,3] and, while the majority of AK lesions remain stable or even regress,[4] studies suggest that 0.6% of AK lesions progress to squamous cell carcinoma (SCC) each year[3] and up to 10% progress over a 10-year period.[5] Anxiety related to the risk of skin cancer may, moreover, reduce health-related quality of life (HRQoL), as may a general unwillingness to interact socially because of the unsightly appearance (particularly when presenting on the face or scalp).[6]

Known risk factors for AK include chronic exposure to the sun (especially sunburn damage), fair skin and blue or light-colored eyes, blond or red hair, advancing age, and immunosuppression.[7-9] In the United States and Europe, these risk factors are common; estimates of the prevalence of AK range from 11% to 26% of the general population.[10,11] The economic burden is substantial; for example, the annual direct costs related to treating AK and the indirect costs of foregone work productivity in the United States were estimated at $1.2 billion and $295 million, respectively, in 2004.[12]

The current treatment options for AK include cryosurgery (removal by freezing), curettage, photodynamic therapy (PDT), and topical creams and gels.[13,14] Physical removal, such as cryosurgery and curettage, is quick and effective on single AK lesions, but may be associated with pain, risk for scarring and high rates of recurrence.[15,16] Physical removal is not practical for treating patients with multiple AK lesions; therefore treatment modalities have been developed to treat the complete area of field cancerization.[15,16] PDT is also quick and effective, but can result in local pain and skin redness following treatment.[16] Topical creams and gels, including imiquimod (both 3.75% and 5%) and diclofenac 3%, have been proven to be effective and may be less painful than physical removal. Treatment durations, however, are long (imiquimod 3.75% requires application once daily for 2 weeks, rest for 2 weeks, and application once daily for 2 weeks; imiquimod 5%, according to the US label, requires application twice weekly for 16 weeks; and diclofenac 3% requires application twice daily for 8-12 weeks[16] and these agents are associated with local skin reactions (LSRs) at the application site, which can lead to poor patient adherence.[17,18]

Ingenol mebutate gel is a novel therapy, which is administered topically for 2 or 3 days and has shown promising results in the treatment of AK. In a pooled analysis of two Phase III trials, ingenol mebutate gel 0.015% applied for 3 consecutive days on the face and scalp demonstrated a 42.2% complete clearance rate at day 57, and LSRs peaked on day 4 (rapidly declining by day 8) and resolved in 2 weeks.[19] After application of the 0.05% ingenol mebutate gel for 2 consecutive days, the corresponding pooled analysis results for complete clearance rate for trunk and extremity lesions assessed at day 57 was 34.1%, and LSRs peaked between days 3 and 8 (rapidly declining thereafter) and resolved by 4 weeks.[19] Because ingenol mebutate gel was well tolerated, there were few discontinuations (2%).[19]

Medical care is expensive and health care budgets are under ever-increasing stress, making it essential that new treatment alternatives be clinically promising and provide good value for money.[20] Effectively allocating resources typically involves estimating the cost-effectiveness of new interventions versus the standard of care.[21] Improvements in patient well-being that are associated with convenience (e.g., shorter treatment duration) and better tolerability (e.g., shorter-lasting adverse events) are difficult to capture in a typical cost-effectiveness analysis. However, a method of capturing patient preferences, that is well-suited for these types of treatment-related benefits and used commonly in dermatology, is willingness-to-pay (WTP)

analysis.[22-29] Specifically, by constructing hypothetical markets (e.g., drug attribute profiles), the analyst solicits (directly or indirectly) the maximum amount a person would be willing to pay, sacrifice or exchange in order to receive a good (e.g., a pharmaceutical) or to avoid something undesired (e.g., an adverse event).

The objective of this study was to estimate the value to potential patients of ingenol mebutate gel's shorter treatment duration and tolerability versus existing topical treatments (imiquimod 3.75%, imiquimod 5% and diclofenac 3%) in the United States.

## MATERIALS AND METHODS

We estimated the incremental WTP in the United States for receiving treatment with ingenol mebutate gel instead of the new generation of topical agents approved by the FDA for AK treatment. Specifically, we considered imiquimod 3.75%, imiquimod 5% and diclofenac 3%, separately, using survey data collected from a panel of subjects with current or previous experience with AK using a web-based questionnaire. The open-ended contingent valuation (CV) survey approach was used due to sample size limitations.[30,31]

### Subjects

Subjects were included if they provided informed consent to participate in the study, resided in the United States, were age 18 years or older, and indicated a current diagnosis of AK. Subjects were recruited from an internet panel representing the U.S. general public (weighted by age, gender, and region) based on a self-reported diagnosis of AK. We re-contacted 67 patients who had participated in a previous omnibus study of AK. Given the small number, e-mail invitations were also sent to 56,800 additional individuals in the panel, and those reporting an AK diagnosis were enrolled in this study. Participants were asked to complete a web-based survey, programmed using the Dub-Quest platform and were estimated to take about 30 minutes to complete. Recruitment began in November 2011 and the survey was open until at least 100 responses were collected (which took 11 days). As the study was non-interventional and the respondents were active members of the online panel with the explicit purpose of participating in web-surveys, ethical approval was not sought.

### Drug Profiles

Product profiles were created for each of the drug agents from the following attributes: (1) method of administration and duration of treatment, (2) expected chance of complete clearance of AK lesions by the end of treatment, (3) treatment-related LSRs (including severity and expected time-to-resolution)[16,32-34] and (4) treatment cost[35] (some or all of which may have been covered by health insurance). Treatment cost for ingenol mebutate gel was not shown in the profile as the subjects were asked about their additional WTP to gain access to ingenol mebutate gel, above and beyond the price of the comparators. See Figure 1 for a full description. To prevent information overload, only the durations of treatment and treatment-related resolution time for LSRs and price per treatment cycle varied across agents.

There is a natural risk of bias in hypothetical choice study designs. Subjects may not accurately report their true preferences (i.e., what they would choose if this was an actual decision). To test internal validity (scope bias), a hypothetical drug profile was included that was less favourable than the imiquimod 5% profile; attributes were identical, but the duration of the LSRs resolution time was considerably longer. The maximum WTP to gain access to ingenol mebutate gel instead of the hypothetical profile, thus, would naturally be greater than the maximum WTP to gain access to ingenol mebutate gel, instead of imiquimod 5%.

**Figure 1.** Treatment Profiles

| Treatment Characteristics | Treatment Profile #1 | Treatment Profile #2 | Treatment Profile #3 | Treatment Profile #4 | Treatment Profile #5 |
|---|---|---|---|---|---|
| **Administration method and treatment duration** | A gel to apply on the affected skin area.<br>Apply once per day, for 2 to 3 days. | A cream to apply on the affected skin area.<br>Apply once per day, 2 days per week, for 16 weeks. | A gel to apply on the affected skin area.<br>Apply twice per day, every day, for 10 weeks. | A cream to apply on the affected skin area.<br>Apply once per day for 2 weeks, rest for 2 weeks, apply once per day for 2 more weeks. | A cream to apply on the affected skin area.<br>Apply once per day, 2 days per week, for 16 weeks. |
| **Expected chance of full clearance of AK lesion by end of treatment** | **Three-quarters (75%)**<br> 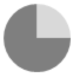 | **Three-quarters (75%)**<br> | **Three-quarters (75%)**<br> | **Three-quarters (75%)**<br> 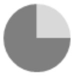 | **Three-quarters (75%)**<br> 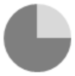 |
| **Treatment-related skin reactions** (redness, flaking/scaling, crusting, swelling, blistering) | Mild to moderate skin reactions<br>Skin reactions clear after 4 weeks. | Mild to moderate skin reactions<br>Skin reactions clear after 18 weeks. | Mild to moderate skin reactions<br>Skin reactions clear after 10 weeks. | Mild to moderate skin reactions<br>Skin reactions clear after 14 weeks. | Mild to moderate skin reactions<br>Skin reactions clear after 30 weeks. |
| **Treatment Cost** (some or all may be covered by health insurance) | | $738 | $448 | $598 | $738 |

AK: actinic keratosis

*Would you be willing to pay extra from your own pocket, in addition to the $Z that may be in part covered by your insurance, to receive access to treatment Profile #X instead of the treatment Profile #Y, even if it was a small amount? Note that the payment could be spread out over a period of 1 year.* ☐ *Yes* ☐ *No*

*What is the maximum amount of money you would be willing to pay to receive the treatment Profile #X instead of the treatment Profile #Y? When you think about this, please bear in mind what you think you would (or would not)* **really** *be prepared to pay, given your actual income and savings. The payment can be spread out over a period of 1 year (i.e., a payment plan).* $______

### WTP Survey

Study subjects were presented with the profiles for ingenol mebutate gel and one of the other agents, side-by-side. Subjects were then asked whether they would be willing to pay an additional amount out-of-pocket to gain access to ingenol mebutate gel instead of the comparator agent to treat their "single most bothersome AK area". As many patients with AK have multiple areas with lesions, often with differing degrees of bothersomeness and prospects for treatment, the study subjects were asked to consider their most bothersome AK area when answering the questions to enhance interpretability. Those who indicated that they were willing to pay extra were then asked their maximum WTP. Because simply asking a respondent to state a maximum WTP often results in a high proportion of zero responses and/or inappropriately large responses, subjects were provided assistance in determining their maximum WTP with a "card-sorting procedure".[30] Subjects were presented with different dollar amounts ($5, $15, $40, $80, $500, $1,000, $3,000) and were asked to evaluate whether they would be "willing to pay", "unwilling to pay", or "unsure" whether they would or would not be willing to pay this amount. The exact wording can be seen in Figure 1. To prevent subjects from being unduly influenced by starting values (i.e., starting point bias), the amounts were presented in random order.[30] This was repeated for each of the four comparator drug profiles (including the hypothetical profile).

The questionnaire also solicited information about patient background and demographics: age; gender; ethnicity; household income; education; employment status and insurance; disease history including current or previous skin cancer, number of current AK lesions, location and size of current lesions, location of previous lesions; and current and previous treatment history of the most bothersome area including treatment-related LSRs following treatment. See Table 1 for specific categories and intervals.

### Statistical Analysis

Subject background and demographic variables were reported as percentages or as means and standard deviations (SDs), as appropriate. Stated WTP was presented as mean and SD. Responses were analyzed for all subjects (where WTP was set to 0 for subjects not willing to pay) and for the sub-group of subjects that had non-zero WTP. The results were also analysed separately by gender, employment status, education level, health insurance, reported current or previous skin cancer, and current or previous experience with different AK treatments.

As noted above, there is a risk that subjects may not accurately report their true preferences. So, the validity of the WTP responses was assessed using well-accepted tests. First, we tested for a positive relationship between household income and WTP (i.e., income effect); lack of an income effect is commonly interpreted as an indication that the subjects did not seriously consider their budget constraint when making hypothetical trade-offs.[36] We conducted this using a multivariate Tobit regression model of the incremental WTP for ingenol mebutate gel, controlling statistically for intervals of household income and a set of background covariates expected to impact WTP (age, gender, health insurance, employment, education, skin cancer experience, size and location of the AK lesion area). As WTP is often skewed (and hence not normally distributed), we repeated the test using logarithmic transformation of the dependent variable. Second, as noted above, we tested for scope bias (i.e., that subjects consistently indicate a higher WTP for "better" profiles) by comparing the estimates of maximum WTP for ingenol mebutate gel rather than the imiquimod 5% profile and hypothetical treatment profile designed to be less favorable. Third, to informally validate that the subjects understood the drug profiles, the survey finished by presenting all five treatment profiles side-by-side (see Figure 1) and by asking the subjects to rank the likelihood that they would successfully complete the full treatment course of each option. Statistical analysis was performed using Stata Statistical Software (Release 11.1. College Station, Texas). Parametric and non-parametric tests were performed using a significance level of 5%.

## RESULTS

### Subjects

A total of 116 subjects completed the survey. Twenty-nine of them were part of the previously identified cohort of AK patients and 87 were members of the internet-based panel. Eleven subjects were excluded because their responses regrding AK diagnosis were contradictory; they reported a diagnosis of AK in the screening question but answered negatively to subsequent questions about having current and previous AK lesions. Two other subjects were excluded because of unrealistic WTP responses; one subject with household earnings between $0 and $19,999 who stated an out-of-pocket WTP of $5,000, and another who gave a maximum WTP of $5,000 in one scenario (imiquimod 5%), but was unwilling to pay for ingenol mebutate gel in the other scenarios (likely misunderstanding the exercise). The final data set included 103 usable responses.

Subject characteristics are described in Table 1. The mean age was 52 years, 64% were women, and 85% were Caucasian. The subjects were generally well-off. While 17% of them reported annual household income of less than $40,000, 56% reported more than $60,000 and 24% more than $100,000. Subjects were also generally well-educated, with 64% reporting highest attainment of a college or university degree and 21% a professional degree. None reported less than high school education. Sixty-three percent of subjects reported active employment, 24% were unemployed, and 13% were retired. Ninety-five percent reported some type of health insurance coverage.

Eighty-one percent of subjects reported having AK lesions at the time of the survey, and 38 patients reported undergoing treatment at the time of the survey. Of these, 71% reported treatment-related LSRs. There were 31% (33% when the two excluded outliers were included) who reported less than full compliance (see Table 1) and the most common reason cited was LSRs.

**Willingness-to-pay**

The WTP results are presented in Table 2. The percentage of subjects indicating a willingness to pay extra for ingenol mebutate gel was highest when the comparator was imiquimod 5% (63%) and lowest when the comparator was the hypothetical profile (48%). Mean WTP for ingenol mebutate gel for those willing to pay something ranged between $475 (vs. diclofenac 3%) and $556 (vs. the hypothetical profile). When subjects who answered that they were unwilling to pay anything were included, the mean incremental WTP ranged between $258 (vs. diclofenac 3%) and $327 (vs. imiquimod 5%).

The results of sub-group analysis are presented in Table 3. Males had higher mean WTP than females, though the difference was not statistically significant. As expected, subjects actively employed had higher WTP for ingenol mebutate gel than those unemployed or retired. Subjects with real-life experience of topical AK treatments had higher mean WTP than those without. Results were statistically significant in the case of ingenol mebutate gel versus imiquimod 3.75% ($820 vs. $339). WTP was also higher (though not statistically significant) for subjects with real-life experience of the relevant treatment described in the profiles. Experience with skin cancer had a small, conflicting, and statistically insignificant effect on WTP. Similarly, experience with LSRs did not have a clear or statistically significant effect on WTP.

**Validation tests**

Despite a limited sample size, there was a clear (though not statistically significant) relationship between household income and increasing WTP for each of the four comparators, suggesting that subjects did consider their budget constraint (see Table 4). Though many were not significant at conventional significance levels, estimates of the other covariates behaved generally as expected (e.g., employment, higher educational attainment, and AK lesions covering the full scalp or full forehead were positively related to WTP) lending further credibility to the results. The percentage of variation explained by the covariates in the regression model (i.e., the Pseudo R2), however, was relatively low, indicating that important factors explaining the WTP for ingenol mebutate gel versus the comparators were not accounted for. The fit was improved when WTP was transformed logarithmically and the Pseudo R2 approximately doubled (see Table 5).

Among those indicating a WTP for ingenol mebutate gel, mean WTP was higher when compared to the less favorable hypothetical profile than in the comparison against the relatively more favorable imiquimod 5% profile, as expected. However, the difference was not statistically significant (see Table 2). The proportion of subjects who were willing to pay extra for the ingenol mebutate gel profile, conversely, was lower when compared with the hypothetical profile than when compared with the imiquimod 5% profile (48% vs. 63%).

This resulted in lower mean WTP for ingenol mebutate gel in the comparison with the hypothetical profile than in the comparison with imiquimod 5% ($327 vs. $265). The difference was statistically significant (p=0.036).

**Table 1.** Subject Characteristics

| | %, mean (SD) |
|---|---|
| **Age (Years)** | 52.2 (15.2) |
| **Men (%)/ Women (%)** | 36/64 |
| **Household Income (%)** | |
| $0 to $19,999 | 6 |
| $20,000 to $39,999 | 11 |
| $40,000 to $59,999 | 25 |
| $60,000 to $99,999 | 32 |
| More than $100,000 | 24 |
| Did Not Answer | 2 |
| **Education and Employment** | |
| Less than high school | 0 |
| High school | 14 |
| College or University Degree (%) | 64 |
| Professional Degree (%) | 21 |
| Other | 1 |
| Employed (%) | 63 |
| **Health Insured (%)** | 95 |
| **Health Insurance** | |
| Private (% of insured) | 64 |
| Medicare (% of insured) | 34 |
| Other Public Insurance (% of insured) | 22 |
| **Current AK** | 81 |
| Current AK: Full face (% of current AK) | 2 |
| Current AK: Full scalp or full forehead (% of current AK) | 16 |
| Current AK: Half scalp or half face (% of current AK) | 42 |
| Current AK: Other (% of current AK) | 40 |
| Current AK: Treatment (% of current AK) | 46 |
| **Current LSRs (% of current AK)** | 32 |
| **Followed current treatment instructions** (% of current AK treatment) | |
| Fully (100%) | 69 |
| Fairly well (75%) | 26 |
| To some extent (50%) | 5 |
| Did not follow (<50%) | 0 |
| **Experience with skin cancer** | 37 |
| **Experience with:** | |
| Topical treatment | 32 |
| Cryosurgery | 34 |
| imiquimod 5% | 9 |
| diclofenac 3% | 14 |
| imiquimod 3.75% | 7 |

Note: Experience was defined as having experienced an event currently, previously or both.
AK: actinic keratosis, SD: standard deviation, LSR: local skin reaction

**Table 2.** Incremental WTP for Treatment with the Ingenol Mebutate Gel Profile instead of Competitor Profiles, US$

| | Vs. Profile #2 (imiquimod 5%) | Vs. Profile #3 (diclofenac 3%) | Vs. Profile #4 (imiquimod 3.75%) | Vs. Profile #5 (hypothetical) |
|---|---|---|---|---|
| % WTP > 0 | 63% | 54% | 50% | 48% |
| Mean (SD) | | | | |
| Only WTP > 0 | 518 (716) | 475 (584) | 514 (645) | 556 (619) |
| Min-max | 25-3500 | 15-3000 | 15-3000 | 25-3000 |
| Mean (SD) | | | | |
| All (censored at 0) | 327 (620) | 258 (491) | 260 (524) | 265 (508) |
| Min-max | 0-3500 | 0-3000 | 0-3000 | 0-3000 |

AK: actinic keratosis, SD: standard deviation, WTP: willingness-to-pay

**Table 3.** Incremental WTP in Subgroups, Subjects willing to pay extra for the Profile of Ingenol Mebutate Gel

| Profile # (corresponding to drug) | Vs. Profile #2 (imiquimod 5%) | Vs. Profile #3 (diclofenac 3%) | Vs. Profile #4 (imiquimod 3.75%) | Vs. Profile #5 (hypothetical) |
|---|---|---|---|---|
| Female | 451 (647) n=39 | 389 (482) n=35 | 356 (354) n=32 | 415 (395) n=29 |
| Male | 619 (812) n=26 | 619 (714) n=21 | 768 (896) n=20 | 762 (813) n=20 |
| p-value | 0.256 | 0.160 | 0.105 | 0.220 |
| Employed | 604 (715) n=47 | 612 (646) n=39 | 629 (611) n=33 | 662 (552) n=32 |
| Not employed <65 years | 430 (864) n=11 | 241 (199) n=10 | 472 (816) n=12 | 483 (811) n=12 |
| Not employed >65 years | 79 (59) n=7 | 47 (8) n=7 | 47 (19) n=7 | 57 (19) n=5 |
| p-value | <0.001 | <0.001 | 0.001 | 0.001 |
| No experience with topical treatment | 311 (255) n=38 | 343 (299) n=35 | 339 (300) n=33 | 378 (329) n=31 |
| Experience with topical treatment | 809 (1010) n=27 | 695 (841) n=21 | 820 (930) n=19 | 863 (856) n=18 |
| p-value | 0.144 | 0.291 | 0.010 | 0.054 |
| No experience with treatment in relevant profile | 504 (676) n=57 | 435 (539) n=48 | 468 (563) n=47 | N/A |
| Experience with treatment in relevant profile | 618 (1011) n=8 | 715 (811) n=8 | 948 (1187) n=5 | N/A |
| p-value | 0.726 | 0.328 | 0.348 | |
| No experience with skin cancer | 452 (500) n=45 | 493 (530) n=39 | 495 (554) n=34 | 523 (455) n=34 |
| Experience with skin cancer | 666 (1056) n=20 | 434 (711) n=17 | 552 (807) n=18 | 633 (903) n=15 |
| p-value | 0.369 | 0.100 | 0.438 | 0.354 |
| Current LSRs | 898 (255) n=24 | 807 (287) n=17 | 775 (214) n=14 | 740 (219) n=14 |
| No current LSRs | 918 (374) n=8 | 1056 (456) n=7 | 913 (401) n=7 | 983 (323) n=6 |
| p-value | 1.00 | 0.90 | 0.91 | 0.54 |

Subjects with WTP>0; AK: actinic keratosis; LSR: local skin reaction; WTP: willingness-to-pay

The results of the third validation test are presented in Figure 2. Almost 90% of the subjects listed the ingenol mebutate gel treatment profile (with the shortest treatment duration and LSR resolution) as the one they were most likely to successfully complete, and 80% listed the hypothetical treatment profile (with the longest duration of treatment and LSRs) as the one least likely to successfully complete.

**Table 4.** Multivariate Tobit Regression of the Determinants of Incremental WTP for Ingenol Mebutate Gel vs. Four Treatment Profiles

| | Vs. Profile #2 (imiquimod 5%) | Vs. Profile #3 (diclofenac 3%) | Vs. Profile #4 (imiquimod 3.75%) | Vs. Profile #5 (hypothetical) |
|---|---|---|---|---|
| **Household Income** | | | | |
| $0 - $19.999 | | Reference Group | | |
| $20.000 - $39.999 | 66.48 | 174.70 | 32.21 | 147.05 |
| $40.000 - $59.999 | 479.37 | 501.39 | 715.45 | 580.40 |
| $60.000 - $99.999 | 282.18 | 279.33 | 747.26 | 526.71 |
| More than $100.000 | 715.44 | 539.96 | 897.53 | 693.99 |
| I choose not to answer | 503.86 | 853.34 | 1136.90 | 764.99 |
| **# of Adults** | 45.57 | 51.82 | -70.69 | -110.37 |
| **# of Children** | 169.78 | 215.74** | 106.34 | 170.56 |
| **Age** | -6.30 | 7.43 | -5.18 | -9.87 |
| **Woman** | -291.33 | -290.57 | -441.35* | -451.70* |
| **Insurance** | 568.91 | -26.51 | -176.54 | -71.87 |
| **Employment** | | | | |
| Employed | | Reference Group | | |
| Not employed <65 years | -90.07 | -349.18 | 58.48 | 251.97 |
| Not employed >65 years | -176.38 | -270.36 | -99.82 | -87.18 |
| **Highest Educational Attainment** | | | | |
| High School | | Reference Group | | |
| College or University | 265.97 | -6.49 | -23.57 | 227.20 |
| Professional Degree | 550.70 | -192.64 | -54.60 | 264.17 |
| Other | 108.73 | 296.58 | 70.46 | 352.89 |
| **Experience of Skin Cancer** | 146.89 | -94.37 | 117.21 | 7.98 |
| **Size and Localization of AK Lesions** | | | | |
| No current AK lesions | | Reference Group | | |
| Current: Full face | -687.94 | -4005.71 | -656.99 | -687.82 |
| Current: Full scalp or full forehead | 673.53* | 726.23** | 405.42 | 319.17 |
| Current: Half scalp or half face | 219.99 | -4.25 | -145.48 | -134.94 |
| Current: Other | 156.66 | 151.89 | -82.27 | -75.13 |
| **Constant** | -1025.97 | -692.94 | 17.68 | 85.33 |
| **Pseudo R2** | 0.0367 | 0.0407 | 0.0254 | 0.0278 |

*p<0.05; **p<0.01; ***p<0.001; AK: actinic keratosis; WTP: willingness-to-pay

**Table 5.** Multivariate Tobit Regression of the Determinants of Incremental WTP for Ingenol Mebutate Gel vs. Four Treatment Profiles – Logarithmally Transformed WTP

| | Vs. Profile #2 (imiquimod 5%) | Vs. Profile #3 (diclofenac 3%) | Vs. Profile #4 (imiquimod 3.75%) | Vs. Profile #5 (hypothetical) |
|---|---|---|---|---|
| **Household Income** | | | | |
| $0 - $19.999 | | Reference Group | | |
| $20.000 - $39.999 | 1.77 | 2.79 | 1.33 | 2.04 |
| $40.000 - $59.999 | 3.98 | 4.72 | 6.05* | 4.95 |
| $60.000 - $99.999 | 2.83 | 3.96 | 6.56* | 4.80 |
| More than $100.000 | 4.20 | 5.47* | 7.55* | 5.58 |
| I choose not to answer | 4.99 | 8.02* | 10.37* | 7.95 |
| **# of Adults** | 0.00 | -0.14 | -0.79 | -0.88 |
| **# of Children** | 1.05* | 1.51** | 0.96 | 1.15 |
| **Age** | -0.03 | 0.05 | -0.02 | -0.03 |
| **Woman** | -1.41 | -1.32 | -1.72 | -1.98 |
| **Insurance** | 3.28 | -0.40 | -1.66 | -1.39 |
| **Employment** | | | | |
| Employed | | Reference Group | | |
| Not employed < 65 years | -0.93 | -1.95 | 0.16 | 1.23 |
| Not employed > 65 years | -0.14 | -0.65 | 0.55 | 0.20 |
| **Highest Educational Attainment** | | | | |
| High School | | Reference Group | | |
| College or University | 0.74 | -0.27 | -1.39 | 0.32 |
| Professional Degree | 2.10 | -1.42 | -1.15 | 0.47 |
| Other | 3.25 | 3.72 | 1.83 | 4.59 |
| **Experience of Skin Cancer** | -0.86 | -1.65 | -0.20 | -1.31 |
| **Size and Localization of AK Lesions** | | | | |
| No current AK lesions | | Reference Group | | |
| Current: Full face | -2.88 | -27.38 | -3.03 | -2.52 |
| Current: Full scalp or full forehead | 1.71 | 1.51 | -0.23 | -0.16 |
| Current: Half scalp or half face | 0.74 | 0.03 | -1.40 | -0.40 |
| Current: Other | 0.88 | 1.22 | -0.66 | -0.32 |
| **Constant** | -3.33 | -3.80 | 0.82 | 0.84 |
| **Pseudo R2** | 0.0931 | 0.0719 | 0.0534 | 0.0437 |

*p<0.05; **p<0.01; ***p<0.001; AK: actinic keratosis, WTP: willingness-to-pay

**Figure 2.** Ranking of Likelihood of Completing Treatments

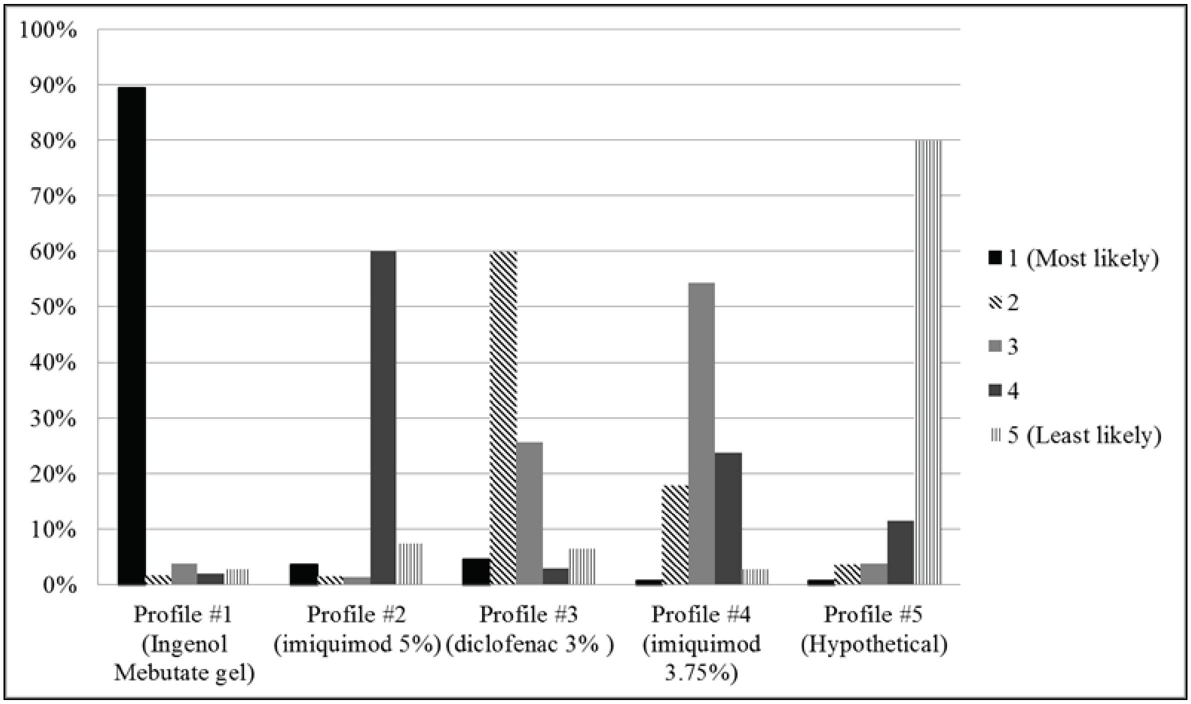

## DISCUSSION

Study results provided consistent evidence of substantial dissatisfaction with three widely used topical treatments for AK. Among subjects undergoing treatment for AK at the time of the survey LSRs were common, and about one-third reported less than full treatment adherence (mostly citing LSRs). A majority of subjects reported a willingness to-pay extra out-of-pocket to gain access to a treatment with shorter duration and shorter-lasting LSRs. Mean stated WTP was large relative to the acquisition prices of the comparator treatments; 44%, 58%, 44% and 36% more for treatment with ingenol mebutate gel in the case of imiquimod 5%, diclofenac 3%, imiquimod 3.75% and the hypothetical profile, respectively. As expected, treatment-experienced subjects were willing to pay more on average than treatment-naïve subjects for shorter treatment duration and shorter-lasting LSRs, given recent real-life experience of LSRs. Exclusion of two subjects with unrealistic WTP responses had a limited effect on the results, for the most part conservatively reducing mean WTP.

Because stated preferences can be less reliable than revealed preferences, we conducted three study validity tests. Despite a relatively small sample size, there was consistent evidence that subjects considered their budget constraint when stating their WTP (i.e., consistent with an "income effect"), especially in the better-fitting logarithmic specification. The results of the internal validation test (bias of scope) were more inconclusive. WTP to avoid treatment with a relatively more favorable profile was paradoxically higher than that to avoid a relatively less favorable profile ($327 vs. $265). This can perhaps best be explained by subject exhaustion; the comparison with imiquimod 5% was first, and the comparison with the hypothetical profile was last (fourth). Subjects may have forgotten the attributes for the imiquimod 5% by the time of the last comparison or perhaps some merely "clicked" through the final scenario (the proportion with non-zero WTP declined with each iteration). Another possible explanation could be that the subjects did not fully understanding the WTP question asked. Nevertheless, the consistency of responses in ranking the likelihood to successfully complete treatment when all five profiles were presented together suggests that subjects understood the profiles, confirming the third validation test.

Important limitations of this study must be acknowledged. Many of the results were not statistically significant, probably due to the small sample size. The estimates did generally behave as expected (within and across scenarios), though, and some estimates nearly achieved statistical significance, suggesting that the study is a good pilot analysis and generally indicative of what can be expected in a larger study. In interpreting the results, one should bear in mind that the subjects may have important differences. The broader population of AK patients in the US data indicates that AK patients in general are relatively old and more likely to be male.[37] In key clinical trials of current topical treatments for AK, for example, mean age ranged from 64 to 67.5 years and the proportion of males ranged from 78-87%.[38-41] The subjects in the current study had mean age of 52 years and 64% were female, reflecting the overrepresentation of women in the panel from which subjects were recruited. Moreover, our subjects were extremely well-educated (85% had a college, university, or professional degree), and household incomes were quite high, perhaps reflecting internet-based recruitment methods. WTP may be higher for these study subjects than for the population of AK patients at large.

While we are unaware of previous estimation of WTP for treatment in the AK setting, it has been used successfully for a variety of dermatologic conditions including psoriasis, atopic eczema, melasma, vitiligo, port wine stains and acne.[22-29] While not generally comparable, the current WTP estimates fall well within the range of estimates observed in other studies (e.g., $125-260 per month for a hypothetical cure for psoriasis, acne, and atopic eczema[22] and single payments of $625-2000 for hypothetical cures of eight different QoL domains individually in psoriasis.[29]

WTP may be a good instrument for capturing patient preferences for dermatological conditions for a number of reasons. First, it is reasonable for most respondents to imagine paying for the treatment of skin diseases because the costs are often manageable (especially when compared to the costs of other important disease areas, such as cancer).[42] Second, WTP does not suffer from "ceiling effects" (i.e., clustering around perfect health when the disease is viewed as "minor"[42]) like other important methods for eliciting patient preferences (including instruments such as the EuroQoL five dimension (EQ-5D) questionnaire and the short form [SF]-36 health survey).

## CONCLUSION

Despite limited sample size, the findings from this study indicate that there is substantial dissatisfaction with current topical agents for AK treatment, and that there is an unmet need for fast-acting topical treatment with short and predictable LSR resolution. This study demonstrates that AK patients have considerable incremental WTP for treatments with shorter durations and shorter-lasting LSRs, which potentially can increase patient adherence to topical treatment.

### Conflict of Interest Declaration

This study was funded by LEO Pharma A/S. Sandra Erntoft is an employee of LEO Pharma A/S. The study was performed by the Swedish Institute for Health Economics (IHE). Michael Willis, Sofie Persson and Ulf Persson are employed at the IHE. Jenny Norlin was employed at IHE at the time of the study, but is currently employed at LEO Pharma A/S.
